# Tumour-infiltrating lymphocytes in oropharyngeal cancer: a validation study according to the criteria of the International Immuno-Oncology Biomarker Working Group

**DOI:** 10.1038/s41416-022-01708-7

**Published:** 2022-01-18

**Authors:** Alhadi Almangush, Lauri Jouhi, Timo Atula, Caj Haglund, Antti A. Mäkitie, Jaana Hagström, Ilmo Leivo

**Affiliations:** 1 Department of Pathology, University of Helsinki, P.O. Box 21, FI-00014 Helsinki, Finland; 2 Research Program in Systems Oncology, Faculty of Medicine, University of Helsinki, Helsinki, Finland; 3 Department of Pathology, University of Turku, Turku, Finland; 4 Faculty of Dentistry, Misurata University, Misurata, Libya; 5 Department of Otorhinolaryngology - Head and Neck Surgery, University of Helsinki and Helsinki University Hospital, P.O. Box 263, FI-00029 Helsinki, Finland; 6 Research Programs Unit, Translational Cancer Medicine, University of Helsinki, P.O. Box 63, 00014 Helsinki, Finland; 7 Department of Surgery, University of Helsinki and Helsinki University Hospital, Helsinki, Finland; 8 Division of Ear, Nose and Throat Diseases, Department of Clinical Sciences, Intervention and Technology, Karolinska Institutet and Karolinska University Hospital, Stockholm, Sweden; 9 Department of Oral Pathology and Radiology, University of Turku, Turku University Hospital, Turku, Finland; 10 Institute of Biomedicine, Pathology, University of Turku, Kiinamyllynkatu 10 D 5035, 20520 Turku, Finland; 11 Turku University Central Hospital, 20521 Turku, Finland

**Keywords:** Head and neck cancer, Prognostic markers

## Abstract

**Background:**

The evaluation of immune response can aid in prediction of cancer behaviour. Here, we assessed the prognostic significance of tumour-infiltrating lymphocytes (TILs) in oropharyngeal squamous cell carcinoma (OPSCC).

**Methods:**

A total of 182 patients treated for OPSCC were included in this study. Assessment of TILs was conducted on tumour sections stained with standard haematoxylin and eosin (HE) staining. We used the scoring criteria proposed by the International Immuno-Oncology Biomarker Working Group.

**Results:**

The multivariable analysis showed that TILs associated with disease-specific survival with a hazard ratio (HR) of 2.13 (95% CI 1.14–3.96; *P* = 0.017). Similarly, TILs associated significantly with overall survival with HR of 1.87 (95% CI 1.11–3.13; *P* = 0.018). In a sub-analysis of HPV-positive and HPV-negative cases separately, TILs showed a significant prognostic value in both groups (*P* < 0.05).

**Conclusion:**

The evaluation of TILs as proposed by the International Immuno-Oncology Biomarker Working Group is a simple and promising method in prediction of survival of OPSCC. It is easily applicable and after further validation can be implemented in the routine pathological report as a basic immune parameter.

## Introduction

Oropharyngeal squamous cell carcinoma (OPSCC) is one of the most common cancers of head and neck region. OPSCC is often associated with human papillomavirus (HPV) infection, but it can also be caused by other risk factors such as tobacco and alcohol abuse. Of note, the incidence of HPV-related OPSCC is increasing rapidly in many countries worldwide [1–5]. Fortunately, patient survival in HPV-related OPSCC is better compared with virus-negative OPSCC, but the estimation of the clinical behaviour of OPSCC is sometimes challenging. Especially with classification of OPSCC as either HPV-positive or HPV-negative there are few additional prognostic factors that can be considered in risk assessment. Adverse prognostic factors include old age, advanced stage and smoking [6]. In daily practice, however, prognostication schemes currently available for OPSCC do not include assessment of the immune status.

Tumour immune microenvironment has been linked strongly with cancer behaviour [7]. For selection of patients regarding treatment strategies, tumour-infiltrating lymphocytes (TILs) have been proposed as biomarkers in many tumour types including those of the head and neck [8]. The International Immuno-Oncology Biomarker Working Group has proposed a method for standardised assessment of TILs in haematoxylin and eosin (HE) stained slides [9, 10] with a good interobserver agreement in a number of studies [11–14]. This method can be considered in the daily practice of the pathologist evaluating TILs [15]. In the present study, we wondered if the local immune cell infiltration in OPSCC could associate with tumour behaviour, and whether it can be measured in routine HE-stained sections. Therefore, we studied a large cohort of OPSCCs including both HPV-positive and HPV-negative tumours, with a sub-analysis to assess the universal use of this prognostic marker.

## Methods

Our cohort included all patients treated for oropharyngeal cancer at the Helsinki University Hospital (Helsinki, Finland) during the 10-year period from January 2000 to December 2009. We excluded patients who had received palliative treatment (*n* = 44), and patients with concurrent head and neck cancers (*n* = 5), with earlier treatments for head and neck cancer (*n* = 11), with histologies other than squamous cell carcinoma (*n* = 18), and cases where tumour tissue was not available (*n* = 71). Tissue samples were collected before radiotherapy or chemoradiotherapy in all but two cases, where post treatment specimens only were available for evaluation. The pretreatment samples included both diagnostic pretreatment biopsies and resected tissues from primary surgery. The patients in this retrospective study were treated between 2000 and 2009. Immunotherapies were not used at that time, so none of the patients received immunotherapy.

A total of 182 cases of OPSCC were included in our analysis of TILs. We used Ventana Inform HPV in situ hybridisation assay to determine HPV status. This study was conducted in compliance with the Declaration of Helsinki and approved by the Research Ethics Committee of the Helsinki University Hospital.

**Fig. 1:**
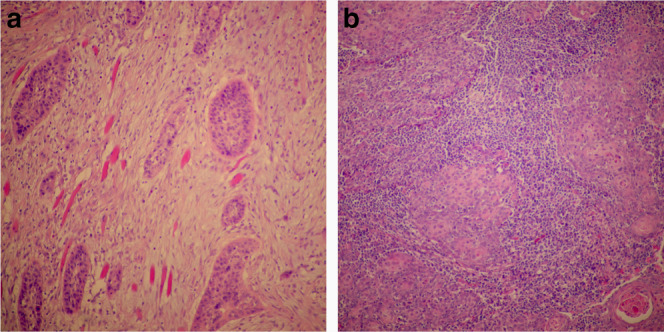
Examples of expression of tumour-infiltrating lymphocytes (TILs) in haematoxylin and eosin-stained sections (magnification ×100) of oropharyngeal squamous cell carcinoma (OPSCC). **a** Scarce expression of TILs in OPSCC where very few immune cells were presented in the stroma. **b** Predominant TILs infiltrate in OPSCC where almost the whole stroma is occupied by TILs.

For the evaluation of TILs, we followed the method introduced by the International Immuno-Oncology Biomarkers Working Group for standardisation of the assessment of TILs in routine HE-stained sections [9, 10]. In brief, the whole slide was scanned at low magnification with ×5 or ×10 objective lens, followed by a higher magnification with ×20 objective lens. Stromal TILs were defined as the percentage of stromal area occupied by infiltrating lymphocytes. The average number of TILs was assessed in multiple stromal areas. Mononuclear immune cells were scored, while polymorphonuclear leucocytes were excluded. In addition, areas of necrosis were excluded. Furthermore, TILs in stromal areas not adjacent to the tumour were excluded. Assessment of TILs was carried out in areas of tumour growth in connective tissues (Fig. 1), while the lymphatic tissue of tonsils was excluded.

All available diagnostic slides stained with H-E were evaluated. TILs were assessed in percentages as a continuous score (5%, 10%, 20%, 30%… etc.). To identify the optimal cutoff point of TIL score with regard to survival, we tested different cutoffs (5%, 10%, 20%, 30% …. etc.) dividing tumours with low TILs and high TILs. Two observers (AA, IL) arranged a training session for the assessment of TILs guided by an experienced head and neck pathologist (IL), and a subsequent reviewing session. Both observers were fully blinded to the clinicopathologic characteristics and the outcome of the cases.

### Statistical analysis

We used IBM SPSS Statistics (version 25) for all statistical analyses. The Kappa Coefficient test was used to evaluate interobserver variance. A two-sided *P* value of <0.05 was considered statistically significant. The relationship between TILs and clinicopathologic characteristics was analysed using cross-tabulation and evaluated by Chi-Square test. We used the Kaplan–Meier estimate and log-rank test for survival analyses. Cox regression was used in univariable and multivariable setting. Multivariable model was used as a method to control confounding factors. Disease-specific survival was measured from the completion of primary treatment to death from disease or last follow-up, while overall survival was measured from the completion of primary treatment to any death or last follow-up. The prognostic value of TILs was analysed separately, and also in combination with T classification as follows:

T1-TILs^High^ includes tumours as described in AJCC 8 (i.e. ≤2 cm in greatest dimension) and TILs are more than or equal to 60%.

T2-TILs^Moderate^ includes tumours as described in AJCC 8 (i.e. >2 cm in greatest dimension but not larger than 4 cm) and with TILs ranging from >20% to <60%.

T3-TILs^Low^ includes tumours as described in AJCC 8 (i.e. >4 cm in greatest dimension or extension to lingual surface of epiglottis) and with TILs less or equal to 20%.

No change in T4 as the tumours in this class severely extend into surrounding tissues.

## Results

The clinicopathologic information of the patients and their relationship with TILs are summarised in Table 1. The cohort included 140 (76.9%) men and 42 (23.1%) women. The median follow-up time was 4.48 years (range 3.51–5.00 years). We assessed stromal TILs because the stroma was the predominant location of TILs in our OPSCC tumours. Infiltration of intra-tumoural TILs was very limited and thus not suitable for a prognostic marker. In the stroma, the expression of TILs ranged from 1 to 90%. No predefined cutoff points were available, but we found 20% as an optimal cutoff point regarding risk stratification of OPSCC. A low infiltration of TILs (i.e. <20%) was found in 49 (26.9%) tumours, while a high infiltration (i.e. TILs ≥20%) presented in 133 (73.1%) tumours. A substantial agreement (Kappa value = 0.78) was found between the two scoring observers. There was a significant association between T-classification and TILs with smaller tumours associating with a higher infiltration of TILs (*P* = 0.01). However, no significant association was noted between TILs and the gender of patients, N-classification, overall stage (I–IV), histological grade, HPV-status, smoking habits, or the treatment regimen given to the patient (*P* > 0.05).

**Table 1:** Association between tumour-infiltrating lymphocytes (TILs) and clinicopathologic characteristics of 182 cases treated for oropharyngeal squamous cell carcinoma.

Variable	Total	Low TILs(<20%)	High TILs(≥20%)	*P* value of chi-square test
	Total, *N* = 182	Number (%)49 (26.9%)	Number (%)133 (73.1%)	
Gender				0.236
Male	140	41 (29.3%)	99 (70.7%)	
Female	42	8 (19%)	34 (81%)	
Smoking				0.538
Never	20	6 (30%)	14 (70%)	
Former	46	10 (21.7%)	36 (78.3%)	
Currently	85	26 (30.6%)	59 (69.4%)	
T classification				**0.010**
T1	35	4 (11.4%)	31 (88.6%)	
T2	68	15 (22.1%)	53 (77.9%)	
T3	40	13 (32.5%)	27 (67.5%)	
T4	39	17 (43.6%)	22 (56.4%)	
N classification				0.473
N0–1	57	13 (22.8%)	44 (77.2%)	
N2–3	125	36 (28.8%)	89 (71.2%)	
Stage				0.644
Early (I–II)	27	6 (22.2%)	21 (77.8%)	
Advanced (III–IV)	155	43 (27.7%)	112 (72.3%)	
Grade				0.34
I	15	3 (20%)	12 (80%)	
II	70	23 (32.9%)	47 (67.1%)	
III	97	23 (23.7%)	74 (76.3%)	
HPV status				0.616
Positive	91	23 (25.3%)	68 (74.7%)	
Negative	91	26 (28.6%)	65 (71.4%)	
Treatment				0.078
Sx ± (C)RT	120	27 (22.5%)	93 (77.5%)	
(C)RT ± Sx	62	22 (35.5%)	40 (64.5%)	

Statistically significant *P* value are in bold.

*Sx* Surgery; *CRT* chemoradiotherapy, *RT* radiotherapy.

**Fig. 2:**
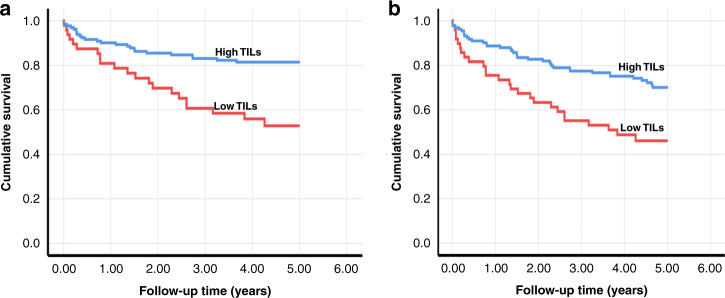
Kaplan–Meier survival curves showing comparison of cases as classified into a low-risk group or a high-risk group based on the expression of tumour-infiltrating lymphocytes (TILs). **a** Disease-specific survival (*P* < 0.001). **b** Overall survival (*P* = 0.001).

The univariable analysis showed a significant association between tumours with low TILs, poor disease-specific survival (HR 2.84, 95%CI 1.58–5.11; *P* < 0.001) and worse overall survival (HR 2.27, 95%CI 1.38–3.75; *P* = 0.001) of the patients. In multivariable models a similar association was observed for both disease-specific survival (HR 2.13, 95%CI 1.14–3.96; *P* = 0.017) and overall survival (HR 1.87, 95%CI 1.11–3.13; *P* = 0.018). Further, Kaplan–Meier curves showed a poor survival for cases with low TILs as shown in Fig. 2 for both disease-specific survival (*P* < 0.001) and overall survival (*P* = 0.001). Interestingly, a significant association of low TILs with poor survival was also seen in both survival analyses (*P* < 0.05) when the cohort was divided into HPV-positive and HPV-negative cases.

Among all clinicopathologic characteristics included in the analyses (Table 2), HPV-status is the only variable that showed a significant association in both disease-specific survival (HR 2.98, 95%CI 1.58–5.64; *P* = 0.001) and overall survival (2.41, 95%CI 1.44–4.05; *P* = 0.001) after all parameters were included in the model. The prognostic value of the other parameters is summarised in Table 2.

**Table 2:** Univariable and multivariable analyses of 182 cases treated for oropharyngeal squamous cell carcinoma.

Factor	Univariable analysis	
	Disease-specific survival	Overall survival
	HR (95%CI); *P* value	HR (95%CI); *P* value
Gender		
Male	1	1
Female	2.19 (0.99–4.88); *P* = 0.054	1.50 (0.85–2.64); *P* = 0.16
Smoking		
Never	1	1
Former	1.66 (0.46–6.05); *P* = 0.44	1.24 (0.48–3.21); *P* = 0.65
Currently	3.29 (1.01–10.7); *P* = 0.048	2.36 (1.01–5.53); *P* = 0.048
T classification		
T1	1	1
T2	1.97 (0.74–5.27); *P* = 0.18	1.92 (0.84–4.43); *P* = 0.12
T3	1.79 (0.62–5.26); *P* = 0.28	2.44 (1.03–5.81); *P* = 0.044
T4	3.62 (1.31–9.96); *P* = 0.013	4.18 (1.79–9.76); *P* = 0.001
N classification		
N0–N1	1	1
N2–N3	2.09 (1.05–4.19); *P* = 0.037	1.49 (0.89–2.48); *P* = 0.129
HPV status		
Positive	1	1
Negative	2.51 (1.38–4.56); *P* = 0.003	2.46 (1.52–3.98); *P* < 0.001
Treatment		
Sx ± (C)RT	1	1
(C)RT ± Sx	1.01 (0.56–1.82); *P* = 0.98	1.13 (0.71–1.81); *P* = 0.604
TILs		
High (≥20%)	1	1
Low (<20%)	2.84 (1.58–5.11); *P* < 0.001	2.27 (1.38–3.75); *P* = 0.001
**Multivariable analysis**		
T classification		
T1	1	1
T2	2.19 (0.79–6.16); *P* = 0.134	1.79 (0.72–4.41); *P* = 0.208
T3	1.49 (0.49–4.43); *P* = 0.478	2.01 (0.77–5.26); *P* = 0.154
T4	2.53 (0.87–7.37); *P* = 0.089	3.48 (1.36–8.87); *P* = 0.009
HPV status		
Positive	1	1
Negative	2.98 (1.58–5.64); *P* = 0.001	2.41 (1.44–4.05); *P* = 0.001
TILs		
High (≥20%)	1	1
Low (<20%)	2.13 (1.14–3.96); *P* = 0.017	1.87 (1.11–3.13); *P* = 0.018

The analyses include overall survival and disease-specific survival for tumour-infiltrating lymphocytes (TILs) and clinicopathologic factors.

When the TIL score was combined with T classification, a total of 25 cases were up-staged from T1-TILs to T2-TILs, and 29 cases were up-staged from T2-TILs to T3-TILs. On the other hand, 16 cases were down-staged from T3-TILs to T2-TILs, and 10 cases from T2-TILs to T1-TILs. Interestingly, a gradual increase in the risk was reported in the analysis of disease-specific survival from T1-TILs to T2-TILs (HR 1.33, 95%CI 0.38–4.68), T3-TILs (HR 2.23, 95%CI 0.65–7.64) and T4 (HR 3.04, 95%CI 0.87–10.68). Similarly, a gradually increased risk was noted in the analysis of overall survival for T2-TILs (HR 1.52, 95%CI 0.52–4.45), T3-TILs (HR 2.06, 95%CI 0.71–6.04) and T4 (HR 3.63, 95%CI 1.24–10.64).

## Discussion

The significance of TILs in predicting cancer outcome has been reported in many studies [8, 16, 17]. A proposal for a standardised method to evaluate TILs in solid tumours using HE-stained sections was introduced recently [9, 10] and a good reproducibility in different cancer types has been reported [11–14]. In the present study, we report the use of this method in assessing TILs in oropharyngeal cancer.

Tumour microenvironment consists of different cell types—including immune cells—that influence cancer progression [18]. Immune response to cancer and the assessment of such a response has been a topic for active research in recent years. Specifically, the evaluation of infiltrating lymphocytes can reveal the status of the pre-existing immunogenicity of the tumour [19]. Similar to previous studies [12, 19–21], we found stromal TILs to be clinically relevant, while intra-tumoural TILs were less important to patient survival. This can be explained by the fact that the immune microenvironment is a major player in tumour-host interactions [22]. The limited prognostic significance of intra-tumoural TILs may be due to the fact that they constitute only a small proportion of total tumour-related TILs. One also needs to appreciate the relative difficulty and inaccuracy in assessment of TILs embedded in intra-tumoural sites in HE-slides [23, 24].

TILs consist of different immune cells (with predominance of T lymphocytes) that have left the blood stream and infiltrated into the tumour tissue and play a major role in the immune response to cancer [25]. Abundance of TILs indicates that antitumour immune response is strong and therefore may contribute to favourable survival [26]. The significance of TILs as a reliable prognostic marker in many tumour types has increased in recent years. The method of overall assessment of TILs in HE-stained sections has shown to be of reliable prognostic value in breast cancer [19], colorectal cancer [14], gastric cancer [27], lung cancer [28], and different subsites of head and neck cancer [8]. In particular, three studies have reported the significance of assessing TILs in HPV-associated OPSCC [29–31] and their findings were in line with our results. However, the method of assessing TILs is not yet standardised for OPSCC. In the present study, we followed the method of the International Immuno-Oncology Biomarker Working Group [9, 10] and found that scoring TILs in HE-stained sections can classify OPSCC tumours into low-risk and high-risk groups. This score is significant for both HPV-positive and HPV-negative OPSCC, demonstrating that TILs can be used as a universal prognostic tool for OPSCC. In our analysis, a high infiltration of tumours by TILs was associated with a better survival in OPSCC, and similar results have been reported in other subsites of head and neck cancer [8], and other cancers as well [14, 19, 32]. The cutoff point of 20% that we identified in this study is similar to those in other studies using the same scoring criteria [12, 33].

To allow for standardised evaluation of TILs in OPSCC, it is important to consider the method that we used in this study, and that has been published in a practical guide for pathologists by the International Immuno-Oncology Biomarkers Working Group with specific recommendations for various locations of cancer including the head and neck [10]. Following this standardised method will accumulate methodologically homogenous data from different populations for future robust meta-analyses. This could allow for a worldwide consensus on the evaluation of TILs in OPSCC. International collaborative efforts are needed to achieve this valuable goal. Of note, such efforts have been undertaken for the assessment of TILs in breast cancer, which has led to an international recommendation [9]. Furthermore, the recent WHO classification of breast tumours recommended the assessment of TILs in daily practice with breast tumours [34]. High reproducibility of results [11–14] and simple technical requirements (just an HE-stained slide) as well as an easy and rapid assessment by pathologists make the assessment of TILs a method of great promise. Therefore, future studies on TILs in OPSCC are advised to follow the method introduced by the International Immuno-Oncology Working Group.

The immune system is important for the efficacy of cancer therapy [19]. Interestingly, accumulated evidence about the clinical significance of assessment of TILs is of major importance. For example, Denkert et al. reported on the use of TILs in predicting response to treatment in breast cancer [19, 35]. Further, Cha et al. [36] reported that TILs scores in core needle biopsies correspond with the status of TILs in resected breast cancer samples. Similar finding was recently reported by Brcic et al. [37] in OPSCC, and therefore further studies are needed to assess the possibility of using TILs in preoperative biopsies and correlating it with treatment response. Reference images for the assessment of TILs in breast cancer are currently available online (www.tilsinbreastcancer.org) and a similar reference for OPSCC would be welcome. Moreover, a digital image analysis of TILs in HE-slides has been reported in many cancers and a significant correlation with the scores of a human observer [14], but with an even better prognostic value [17]. Such computer-based assessment of TILs should be part of future studies assessing TILs in OPSCC.

Although HPV + OPSCCs have a better survival rate than HPV-negative cases, some cases of HPV+ OPSCCs may have an aggressive behaviour [38]. It is of great clinical importance to recognise those HPV+ OPSCC cases with good prognosis and therefore eligible to de-escalation therapy (i.e. less intensive treatment with elimination of chemotherapy and/or reduction in radiation [39]). Indeed, successful de-escalation requires an accurate risk-stratification. Findings of the present study indicate that those HPV+ OPSCC patients who could benefit from de-escalation may be identified by assessing their immune response to cancer cells. Our study reports that the TIL score is a good representative of an immune response which significantly relates to patient survival, as also reported elsewhere [40, 41]. We suggest that assessment of TILs should be included in pathology reports and considered in clinical risk stratification of OPSCC. TILs score in OPSCC can be considered for upstaging (in tumours with low TILs) or downstaging (in tumours with high infiltration of TILs). Incorporation of TILs in the TNM classification could be a step towards the introduction of TNM-Immune, as has been considered recently in some cancers [42–44], but not yet in OPSCC. Therefore, future studies with larger multi-institutional cohorts are necessary.

Indeed, to reach a more precise prognostication it is important to take multiple prognostic factors into consideration including different aspects such as immune-related, cancer-related, and patient-related. TILs score should be considered when deciding the need for adjuvant therapy of OPSCC. In addition, assessment of TILs may also serve as a predictive marker in assessing treatment response in OPSCC cases. Furthermore, evaluation of TILs may be considered in ongoing immunotherapy trials in head and neck cancer [45]. Such prospective clinical trial datasets have high accuracy and reliability for validation of TILs as a biomarker [46]. Interestingly, clinical trials in breast cancer immunotherapy have reported prognostic significance of TILs in HE-stained sections [46, 47]. Similar evaluations of TILs in trials of head and neck cancer are required.

In conclusion, the assessment of TILs using readily available HE-stained sections is a cost-effective tool that can be used as an immune-based classification for OPSCC. Limitations of our present findings include the retrospective nature of the study, and the fact that it was based on a single-institution cohort. Of note, our findings are supported by recent studies on cancers of the oropharynx [40, 41] and other subsites of the head and neck [12, 23, 48], as well as other locations [14, 19, 32] reporting prognostic usefulness of TILs in HE-stained sections. In addition, the method of assessment used in this study is well-defined and reported to yield good reproducibility and reliability as a prognostic marker in various cancers [11, 14]. Recent research has also confirmed the clinical significance of assessing TILs in OPSCC [49] and other cancers [50, 51]. Therefore, the method used in this study can be considered as a standardised method for further validations in other cohorts of OPSCC to allow future implantation of TILs in daily practice.

## Data Availability

All data that reported in this study is available from the corresponding author on reasonable request.
